# Alterations in resting‐state whole‐brain functional connectivity pattern similarity in bipolar disorder patients

**DOI:** 10.1002/brb3.2580

**Published:** 2022-04-21

**Authors:** Liangliang Ping, Cong Zhou, Shan Sun, Wenqiang Wang, Qi Zheng, Zhiyi You

**Affiliations:** ^1^ Department of Psychiatry Xiamen Xianyue Hospital Xiamen China; ^2^ School of Mental Health Jining Medical University Jining China

**Keywords:** bipolar disorder, FcHo, functional connectivity, resting state

## Abstract

**Background:**

Previous neuroimaging studies have extensively demonstrated many signs of functionally spontaneous local neural activity abnormalities in bipolar disorder (BD) patients using resting‐state functional magnetic resonance imaging (rs‐fMRI). However, how to identify the changes of voxel‐wise whole‐brain functional connectivity pattern and its corresponding functional connectivity changes remain largely unclear in BD patients. The current study aimed to investigate the voxel‐wise changes of functional connectivity patterns in BD patients using publicly available data from the UCLA CNP LA5c Study.

**Methods:**

A total of 45 BD patients and 115 healthy control subjects were finally included and whole‐brain functional connectivity homogeneity (FcHo) was calculated from their rs‐fMRI. Moreover, the alterations of corresponding functional connectivity were subsequently identified using seed‐based resting‐state functional connectivity analysis.

**Results:**

Individuals with BD exhibited significantly lower FcHo values in the left middle temporal gyrus (MTG) when compared with controls. Functional connectivity findings further indicated decreased functional connectivities between left MTG and cluster 1 (left superior temporal gyrus, extend to middle temporal gyrus, rolandic operculum), cluster 2 (right postcentral, extend to right precentral) in BD patients. The mean FcHo values of left MTG were positively correlated with insomnia, middle scores and appetite increase scores. The mean functional connectivities of left MTG to cluster 1 were negatively correlated with grandiose delusions scores. While the functional connections between left MTG with cluster 2 were negatively correlated with delusions of reference and positively correlated with insomnia, middle scores in BD patients.

**Conclusions:**

Our findings suggested that abnormal FcHo and functional connections in those areas of the brain involving DMN and SMN networks might play a crucial role in the neuropathology of BD.

## Graphical abstract

A total of 45 BD patients and 115 healthy control subjects were finally included and whole‐brain functional connectivity homogeneity (FcHo) was calculated from their rs‐fMRI. Seed‐based resting‐state functional connectivity analysis was then used to reveal the alteration of corresponding functional connectivity. Our findings indicated that whole‐brain FcHo and connectivity abnormalities of left middle temporal gyrus might play a crucial role in the neuropathology of BD.

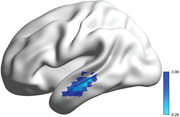

## 1 INTRODUCTION

Bipolar disorder (BD) is a complex, severe, and chronic mental disorder characterized by alternating periods of manic or hypomanic episodes and depressive episodes (Carvalho et al., 2020). The suicide rates among individuals with BD are approximately 20–30 times higher than the rates in the general population (Miller & Black, 2020). Unfortunately, the specific neurophysiologic basis of BD is unknown. In recent years, as a promising neuroimaging measure, resting‐state functional magnetic resonance imaging (rs‐fMRI) can detect blood oxygen level‐dependent (BOLD) signal during rest to assess brain function and pinpoint the relation between the altered spontaneous neural activity of specific brain regional and physiological states (Biswal et al., 1995; Fox & Raichle, 2007). Due to its noninvasiveness and high spatial and temporal resolution, rs‐fMRI has been applied to investigate psychiatric disorders (Canario et al., 2021). For several commonly used methods, the amplitude of low‐frequency fluctuation (ALFF) (Zang et al., 2007) measures voxel‐wise fluctuations in the total power of the BOLD signal at very low frequencies (typically 0.01−0.10 Hz). Regional homogeneity (ReHo) (Zang et al., 2004) characterizes the relationship between the time series of a voxel with nearby voxels using Kendall's coefficient of concordance. Functional connectivity (FC) is a measure that correlates the time series of two different spatial regions in the brain with the assistance of linear temporal correlation (Smitha et al., 2017). Based on measuring ALFF, ReHo, and/or the FC of rs‐fMRI, a large body of neuroimaging studies has extensively demonstrated many signs of functionally spontaneous local neural activity alterations in individuals with BD compared to healthy controls (Gong et al., 2021; Qiu et al., 2019; Shan et al., 2020; Syan et al., 2018; Wang et al., 2020b; Whittaker et al., 2018; Xi et al., 2021; Xiao et al., 2019; Yu et al., 2019; Zhang et al., 2019; Zhong et al., 2019). Although these investigations have provided valuable insights into the dysfunctions of the brain in BD, it remains mostly unknown how to detect alterations in the pattern of voxel‐wise whole‐brain functional connectivity. Thus, assessing the voxel‐wise whole‐brain functional connectivity pattern similarity is beneficial in providing information to identify the pathophysiology of BD.

Wang et al. (2018) recently developed the whole‐brain functional connectivity homogeneity (FcHo) approach, which effectively evaluates the homogeneity of a given voxel's whole‐brain functional connectivity pattern with 26 voxels of its nearest neighborhood rather than counting the number of connections. In comparison to other commonly used approaches, this way can better characterize the similarity of voxel‐wide whole‐brain functional connectivity patterns rather than the similarity of functional activity and regional functional connectivity, as well as better define association cortical areas and high‐order cognitive brain regions with higher FcHo values, while primary sensory and motor related areas with lower FcHo values, without the need to choose the thresholds of connectivity strength for calculating connectivity strength.

Additionally, some researchers have utilized this approach to examine functional anomalies and the mechanism of electroconvulsive therapy in depression (Wang et al., 2020a; Wang et al., 2019).

In the current study, to show aberrant functional connectivity patterns in BD patients, we used the FcHo method on resting‐state fMRI data from a publically available data set. Furthermore, resting‐state functional connectivity was also used to determine the most important connections which mostly contributed to the FcHo changes in the patients with BD.

## 2 MATERIALS AND METHODS

### 2.1 Participants

All data included in this work were acquired from the UCLA CNP LA5c Study, which is openly accessible via the OpenfMRI database (https://openfmri.org/dataset/ds000030/) (Poldrack et al., 2013). A broad sample of demographic, clinical, and multimodal neuroimaging data is included in CNP dataset. All of the subjects were right‐handed and aged 21−50. The Structured Clinical Interview for the Diagnostic and Statistical Manual of Mental Disorders Fourth Edition‐Text Revision (DSM‐IV) was used to establish diagnoses. The data descriptor report contains comprehensive descriptions of participant recruitment, exclusions, and research strategies (Poldrack et al., 2016). All individuals provided written informed agreement in accordance with protocols authorized by the UCLA and Los Angeles County Departments of Mental Health Institutional Review Boards. After screening and quality control of the data, we finally excluded 19 participants, of which 8 missed imaging data and 10 experienced excessive head movement (translational or rotational motion parameters greater than 3 mm or 3° during the fMRI scan), and 1 had wrongs during fMRI preprocessing.

The clinical symptoms of individual patients were evaluated using the Scale for the Assessment of Negative Symptoms (SANS) and the Scale for the Assessment of Positive Symptoms (SAPS) (Andreasen, 1990). To determine the severity of depressive symptoms, the 28‐item Hamilton Rating Scale for Depression (HAMD) was employed (Hamilton, 1960). The 11‐item Young Mania Rating Scale (YMRS) was applied to measure the severity of manic symptoms (Young et al., 1978).

The demographic characteristics of the sample finally included in this study, which included 115 healthy volunteers and 45 bipolar disorder type I (BD) patients, is shown in Table 1.

**TABLE 1:** Demographics and clinical characteristics of the study's participants (mean ±standard deviation [SD])

Subjects	BD (*n* = 45)	HC (*n* = 115)	*X* ^2^/*t*	*p*
Number of subjects	45	115		
Gender (male/female)	26/19	62/53	0.070	.791
Age (years)	35.02 ± 9.01	31.07 ± 8.62	2.576	.011
Education level (years)	14.64 ± 1.99	15.13 ± 1.66	−1.574	.118
Mean FD (mm)	0.11 ± 0.06	0.09 ± 0.05	1.514	.132
SAPS	8.47 ± 8.59	–	–	–
SANS	20.56 ± 13.68	–		–
YMRS	11.51 ± 10.64	–	–	–
HAMD	18.82 ± 13.44	–	–	–

*Note*: A Pearson's chi‐square test (*X* ^2^) was used for gender comparison. Two‐sample *t*‐tests (*t*) were used for age, education comparisons. The data are presented as the mean ± standard deviation.

Abbreviations: BD, bipolar disorder; FD, frame‐wise displacement; HAMD, the 28‐item Hamilton Rating Scale for depression.; HC, healthy controls; SANS, the scale for the assessment of negative symptoms; SAPS, the scale for the assessment of positive symptoms; YMRS, the 11‐item Young Mania Rating Scale.

### 2.2 MRI data acquisition

The functional and anatomical MRI data of all participants were collected on the 3T Siemens Trio scanner. Resting‐state functional images data were collected using a T2*‐weighted echo planar imaging sequence (repetition time/echo time = 2000/30 ms, voxel size = 3 mm × 3 mm × 4 mm, flip angle = 90°, FOV = 192 mm × 192 mm, matrix = 64 × 64, slice thickness = 4 mm, 34 slices, and 152 volumes, oblique slice orientation). The parameters of high‐resolution structural MPRAGE images were as follows: repetition time/echo time = 1900/2.26 ms, FOV = 250 mm × 250 mm, matrix = 256 × 256, slice thickness = 1 mm, 176 slices, sagittal plane.

### 2.2 Data preprocessing

All resting‐state fMRI data were preprocessed using DPARSF (http://rfmri.org/DPARSF) software (Yan et al., 2016). The first 10 volumes of were discarded. The subsequent steps included slice timing, realign, head motion correction, normalization of the images to echo‐planar imaging (EPI) template in Montreal Neurological Institute (MNI) space (resampled to voxel size of 5 × 5 × 5 mm^3^ to reduce computation time), smoothing with a Gaussian kernel of 6 mm full width at half‐maximum (FWHM). Following that, regressing out nuisance covariates including Friston‐24 parameters of head motion, white matter and cerebrospinal fluid signal. Finally, data were linearly detrended and filtered with a temporal band‐pass (0.01−0.1 Hz). Ten subjects (HC = 6, BD = 4) were excluded because their head motion >3 mm or rotation >3°in any direction.

Furthermore, before performing functional connectivity analysis, the fMRI data were preprocessed according to the following steps: normalized to EPI template, resampled to 3 × 3 × 3 mm^3^ voxel size, and smoothed with a Gaussian kernel of 6 mm FWHM. Taking into account that the regression of the whole‐brain signal would exaggerate the anticorrelation, we did not perform it in order to ensure the reliability of results.

### 2.3 Voxel‐wise whole‐brain FcHo analyses

FcHo was calculated for each voxel via Kendall's coefficient concordance (KCC) to quantify the similarity of the whole‐brain connectivity pattern. The detailed description of the calculation formula can be found in the corresponding article (Wang et al., 2018).

Finally, each subject's whole‐brain FcHo map was generated for statistical analysis. A two‐tailed two‐sample *t*‐test was conducted to reveal the disrupted whole‐brain FcHo differences between patients compared to healthy controls, using gender, age, years of education, and average FD as covariates. The threshold for significance was set at voxel *p* < .0001 and cluster *p* < .001 (Gaussian random field correction).

### 2.4 Functional connectivity analyses

In this part, those regions that exhibited significant alterations in FcHo in BD patients were defined as seed regions. These brain areas were then employed to perform a voxel‐wise whole‐brain functional connectivity analysis to further highlight key functional connectivity. First, we resampled the seed regions’ masks into a voxel size of 3 × 3 × 3 mm^3^ and extracted the mean time series. Second, we applied Pearson's correlation coefficient to estimate functional connectivity between the seed region's averaged time series and remaining brain voxels. Then fisher's *z* transformation was performed to convert functional connectivity to *z* value. With gender, age, years of education, and mean FD as covariates, a two‐sample *t*‐test was conducted to detect statistical difference in functional connectivity between BD and HC. The significance was determined using the Gaussian random field (GRF) theory multiple comparison corrections (voxel *p* < .001, cluster *p* < .01, two‐tailed). The names and cluster sizes of significant brain regions were recorded based on the automated anatomical labeling (AAL) atlas (Tzourio‐Mazoyer et al., 2002). The results were visualized with BrainNet Viewer (http://www.nitrc.org/projects/bnv/) (Xia et al., 2013).

### 2.5 Correlation analyses

Spearman's correlation analysis was performed to examine the association between neuroimaging indicators (mean FcHo and mean functional connections) and clinical characteristics including age, the total score and all the subitems score of SAPS, SANS, HAMD, and YMRS. The significance level was set at *p* < .05.

## 3 RESULT

### 3.1 Demographics and clinical characteristics

The demographics and clinical characteristics of the sample in this study are shown in Table 1. We found no significant differences in gender (*X* ^2 ^= 0.070, *p* = .791), education level (*t* = −1.574, *p* = .118) except for the age (t = 2.576, *p* = .011) between the BD and HC groups.

### 3.2 Changed FcHo between BD and HC groups

**FIGURE 1:**
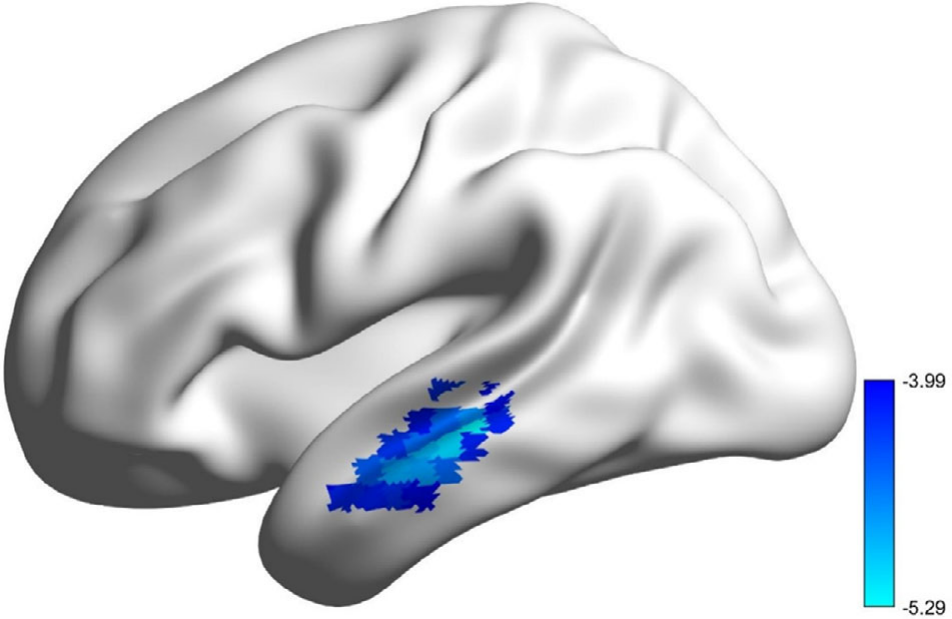
Reduced whole‐brain functional connectivity pattern homogeneity (FcHo) of left middle temporal gyrus (MTG) in BD patients. The significant threshold was set at voxel *p* < .0001 and cluster *p* < .001 (two‐tailed) using Gaussian random field (GRF) correction

When compared with healthy, patients with BD showed significantly reduced FcHo in the left middle temporal gyrus (MTG) (peak MNI coordinate: −60 −26 −7, size of voxels: 6000 mm^3^) (Figure 1).

### 3.3 Changed functional connectivity

**FIGURE 2:**
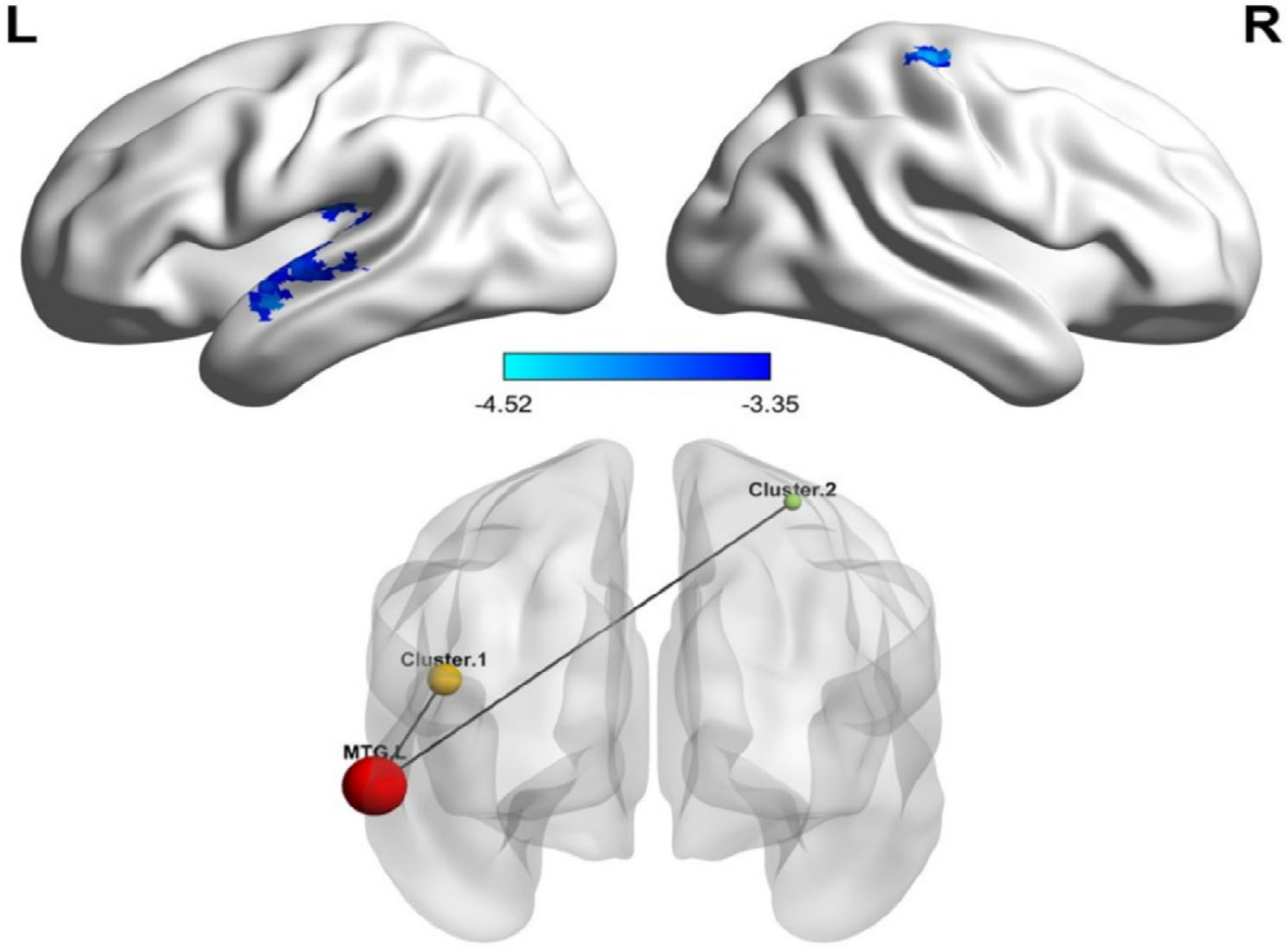
Altered resting‐state functional connectivities in BD patients. Seed‐based functional connectivity analyses showed decreased functional connections between left MTG and cluster 1 (left superior temporal gyrus, extend to middle temporal gyrus, rolandic operculum), cluster 2 (right postcentral, extend to right precentral) in BD patients. The significant threshold was set at voxel *p* < .001 and cluster *p* < .01 (two‐tailed) using GRF correction

Significantly decreased functional connectivities were described between left MTG and cluster 1 (left superior temporal gyrus, extend to middle temporal gyrus, rolandic operculum), cluster 2 (right postcentral, extend to right precentral) in BD patients compared with HC (Table 2 and Figure 2).

**TABLE 2:** Abnormal brain function regions in BD patients

			Peak coordinate			
	Brain regions	Cluster Size (mm^3^)	*X*	*Y*	*Z*	*t* Values
FcHo	Left MTG	6000	–60	–26	–7	–5.29
FC	Left STG extend to left MTG, left rolandic operculum	4509	–45	–18	18	–4.30
	Right postcentral, extend to right precentral	3672	30	–27	60	–4.38

Abbreviations: MTG, middle temporal gyrus; STG, superior temporal gyrus.

### 3.4 Correlation analyses

**FIGURE 3:**
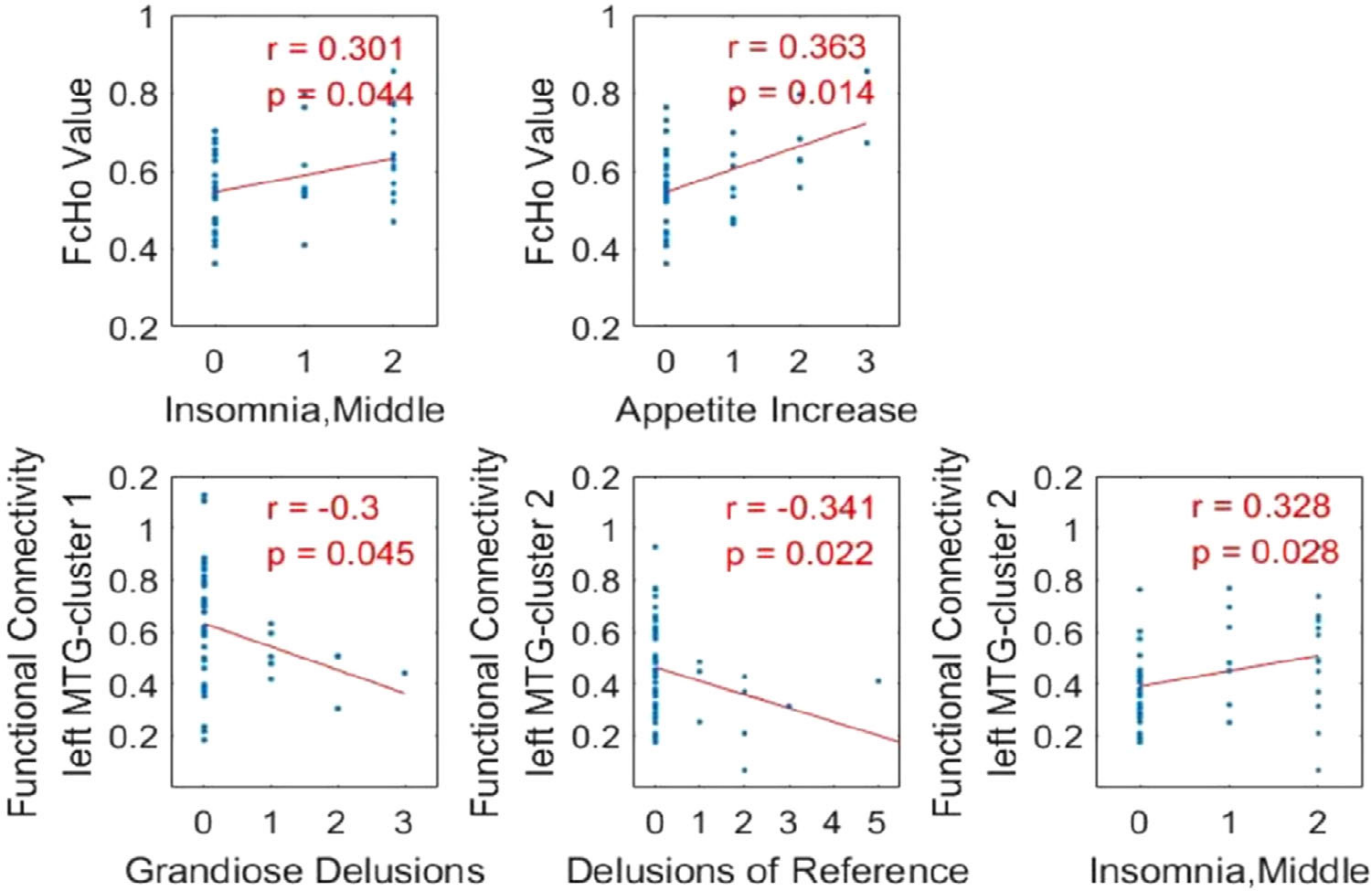
Spearman's correlation analyses between neuroimaging metrics and clinical characteristics. The upper part of the figure showed correlation analyses identified a positive correlation between FcHo values in left MTG and the HAMD subitems insomnia, middle scores, and appetite increase scores in the BD group. The lower part of the figure showed correlation analyses identified a negative correlation between the functional connectivity of left MTG with cluster 1 and SAPS subitems grandiose delusions, a negative correlation between the functional connectivity of left MTG with cluster 2 and SAPS subitems delusions of reference, and a positive correlation between the functional connectivity of left MTG with cluster 2 and the HAMD subitems insomnia, middle scores in BD patients. The significance was set at *p* < .05 (uncorrected)

For mean FcHo values of left MTG and functional connectivities of left MTG with cluster 1 and cluster 2, we performed a Spearman's correlation analysis to investigate their association with the clinical variables such as age and the scores of SAPS, SANS, HAMD, and YMRS in the BD group. No significant correlation between mean FcHo values and functional connectivities in the anatomical regions with age, the total scores of SAPS, SANS, HAMD, and YMRS were found in BD patients (all *p* > .1) (see Table S1 in supplementary material). We found that mean FcHo values of left MTG were significantly positively correlated with the HAMD subitems insomnia, middle scores (*R* = 0.301, *p* = .044) and appetite increase scores (*R* = 0.363, *p* = .014) in patients with BD (Figure 3). In addition, the mean resting‐state functional connections of left MTG to cluster 1 were negatively correlated with the SAPS subitem grandiose delusions (*R* = −0.30, *p* = .045). The functional connections between left MTG with cluster 2 were negatively correlated with the SAPS subitem delusions of reference (*R* = −0.341, *p* = .022) and positively correlated with the HAMD subitems insomnia, middle scores (*R* = 0.328, *p* = .028) in BD patients (Figure 3). None of the nominally significant relationships survived multiple comparisons (*p* > .05, FDR corrected).

## 4 DISCUSSION

In contrast to previous fMRI studies of BD, the present research employed a newly developed fully data‐driven FcHo approach that did not rely on any hypothesis to explore whole‐brain functional connectivity patterns in patients with bipolar disorder. And we adopted seed‐based functional connectivity to identify functional brain deficits in BD and HC. Individuals with BD showed lower FcHo in the left MTG compared to healthy controls. Seed‐based functional connectivity analysis identified significantly reduced FC between left MTG with multiple brain areas linked in the default mode network (DMN) and the sensorimotor network (SMN) (including left STG, left MTG, left rolandic operculum, right postcentral, right precentral) in BD patients group. Moreover, follow‐up correlation analysis revealed multivariate associations between the changed neuroimaging measurements and some clinical characteristics such as insomnia, middle scores, appetite increase scores grandiose delusions scores, and delusions of reference scores.

In our current study, decreased FcHo was revealed in the left MTG. This finding was strongly supported by a growing body of prior neuroimaging studies, which have broadly revealed that BD participants had both morphological and functional abnormalities of left MTG (Chai et al., 2020; Chrobak et al., 2021; Hibar et al., 2018; Liu et al., 2021; Wang et al., 2016). The MTG involves in the processing of numerous complex emotions, cognitions, and behaviors (Xu et al., 2019). In addition, a recently meta‐analytic study (Smallwood et al., 2021), generated using the Neurosynth (Yarkoni et al., 2011) database (https://neurosynth.org/), suggests that the DMN, as defined by REF (Yeo et al., 2011), was engaged across multiple features of human cognition (including episodic, linguistic, social, and emotional). In the meantime, the left middle temporal cortex (MTC), regarded as a hub of the DMN, seems to show the most specific function profile because it is only implicated in linguistic and social. Furthermore, correlation analysis revealed that the mean FcHo value of the left MTG in BD was associated with moderate insomnia and increased appetite in the HAMD subitems. According to a recent study that used voxel‐wise degree centrality (DC) analysis, patients with primary insomnia exhibited lower DC values in the left MTG than healthy control subjects (Yan et al., 2018). Qin et al. (2021) demonstrated that the left MTG participated in the formation of a consciousness modulation circuit using an approach that combined degree centrality graph‐theoretical assessment and regions of interest (ROI)‐based functional connectivity. These findings support the theory that insomnia is linked to alterations in MTG function. Evidence of a meta‐analysis has confirmed the grey matter volume (GMV) reductions of the left middle temporal cortex in obese patients (Herrmann et al., 2019). Additionally, a recent study also suggested that BD patients showed a relationship between weight gain and left middle temporal gyrus volume loss (Bond et al., 2019). Taken together, these findings suggest that abnormal neuronal spontaneous activity in left MTG may be a biological marker of BD.

In addition, we noticed the vital connections that contribute significantly to the alteration in the similarity of the whole‐brain functional connection pattern in BD, were mainly involved in the DMN and SMN areas. Moreover, as shown in Figure 2, abnormal FC in the DMN and the SMN were negatively correlated with delusions symptoms. Previous evidence has shown that BD patients had disrupted connectivity in the DMN (Gong et al., 2021; Wang et al., 2020b; Wang et al., 2020c) and SMN (Doucet et al., 2017; Martino et al., 2016; Wang et al., 2020c). Moreover, Martino et al. (2016) reported DMN‐SMN imbalance with frequency‐specific resting‐state variability in bipolar depression and mania states. By examining whole‐brain dynamic functional connectivity (dFC), Liu et al. (2021) also found aberrant dFC in the brain areas associated with the DMN and SMN across mood states in BD. Taken together, these overall results, both with our findings of aberrant functional connectivity between the DMN and SMN, suggest that network integration and segregation may be damaged in BD, leading to diverse clinical manifestations. These findings could greatly help improve our knowledge of the mechanisms of BD.

## 5 LIMITATIONS

The current research has several limitations that should be properly considered. First, the number of individuals in each group is relatively small, and due to the lack of an independent data set, additional external validation of the current findings was not performed. Therefore, further studies should be extended in a larger, more diverse transdiagnostic sample. Second, the use of medications and/or the chronic duration of the illness in the patient groups may have an influence on the present findings of this study. In the future, it will be important and essential to undertake investigations on wit drug‐naive first‐episode patients.

## 6 CONCLUSION

In conclusion, our investigators showed reduced whole‐brain FcHo in the left MTG, as well as aberrant resting‐state functional connectivities between the left MTG and multiple brain regions related to the DMN and SMN in BD patients. These findings revealed a disruption in the integration of semantic and episodic processing in BD patients. It further highlighted the importance of DMN‐SMN dysfunction in the neuropathology of BD.

## CONFLICT OF INTEREST

All authors declare no any potential conflicts of interest.

### PEER REVIEW

The peer review history for this article is available at https://publons.com/publon/10.1002/brb3.2580.

## Supporting information

Supporting information

## Data Availability

The data that supports the findings of this study is publicly available via the OpenfMRI database (https://openfmri.org/dataset/ds000030/).
