# Supplementary material for: Alterations in resting‐state whole‐brain functional connectivity pattern similarity in bipolar disorder patients
Source: Brain Behav. 2022 Apr 21;12(5):e2580. doi: 10.1002/brb3.2580 (PMC9120726; doi:10.1002/brb3.2580)
Supplement: Supplementary file 1 — Supporting information [file BRB3-12-e2580-s001.docx]

**TABLE S1** Correlations between mean FcHo values and functional connectivities in the anatomical regions and clinical variables in BD group

| Brain regions | | age | | SAPS | | SANS | | YMRS | | HAMD | |
| --- | --- | --- | --- | --- | --- | --- | --- | --- | --- | --- | --- |
|  |  | R | p | R | p | R | p | R | p | R | p |
| FcHo | left MTG | -0.067 | 0.663 | 0.118 | 0.439 | -0.127 | 0.407 | 0.229 | 0.131 | 0.015 | 0.921 |
| FC | Cluster 1 | -0.064 | 0.676 | 0.067 | 0.661 | -0.159 | 0.296 | 0.150 | 0.326 | -0.064 | 0.677 |
|  | Cluster 2 | -0.025 | 0.870 | -0.044 | 0.775 | 0.123 | 0.420 | 0.022 | 0.885 | 0.060 | 0.696 |

Note: The R and p values were obtained using Spearman correlation analysis.

Abbreviations: SAPS, the scale for the assessment of positive symptoms; SANS, the scale for the assessment of negative symptoms; YMRS, the 11-items Young Mania Rating Scale; HAMD, the 28-items Hamilton Rating Scale for depression.; MTG, middle temporal gyrus
